# Can Surface Coating of Circular Saw Blades Potentially Reduce Dust Formation?

**DOI:** 10.3390/ma14185123

**Published:** 2021-09-07

**Authors:** Roman Myna, Raphaela Hellmayr, Maria Georgiades, Lena Maria Leiter, Stephan Frömel-Frybort, Rupert Wimmer, Falk Liebner

**Affiliations:** 1 Institute of Wood Technology and Renewable Materials, University of Natural Resources and Life Sciences, Konrad Lorenz Straße 24, 3430 Tulln, Austria; roman.myna@boku.ac.at (R.M.); raphaela.hellmayr@boku.ac.at (R.H.); mgeorgiades@groupwise.boku.ac.at (M.G.); lena.leiter@boku.ac.at (L.M.L.); 2 Kompetenzzentrum Holz GmbH, Altenberger Straße 69, 4040 Linz, Austria; stephan.froemel-frybort@htl.moedling.at; 3 Institute of Chemistry of Renewable Resources, University of Natural Resources and Life Sciences, Konrad Lorenz Straße 24, 3430 Tulln, Austria; Falk.Liebner@boku.ac.at

**Keywords:** wood dust, electrostatic charging, triboelectric effect, saw blade coating, electrical field strength

## Abstract

Coating of steel is a frequently applied approach to increase the resistance of moving machine parts towards abrasion, surface oxidation, and corrosion. Here, we show that plating circular saw blades with certain metals can help to reduce the electrical charging of wood dust during cutting, which has significant implications for occupational safety, healthcare, and lifetime of filter systems. With the example of beech wood planks, machine net energy consumption *E*_V_ (J cm^−3^) and cumulated field strength ${\vec{E}}_{V}$ (kV m^−4^) as caused by electrically charged particles were compared for cutting of 10- and 20-mm deep grooves (800 mm length) using saw blades of different toothing (24, 60 teeth) and surface coating (Cu, Ag, and Cr). To ensure uniform feed per tooth (*f_z_* = 0.063 mm), saw blades were operated at different rotation speeds (4000 vs. 1600 rpm). The results demonstrate that the extent of electrostatic sawdust charging can be manipulated to a certain extent by the type of saw blade plating. Coating with chromium turned out to be most effective in shifting the electrostatic charge of the wood particles towards neutralization. Lowering of rotation speed using circular saw blades of higher toothing was an additional measure significantly reducing electrostatic charging of wood dust. Hence, cutting with a chrome-coated blade with 60 teeth can be specifically recommended as the reduction of electrical saw dust charging is not associated with higher machine power consumption.

## 1. Introduction

Wood harvesting, processing, and utilization is almost as old as human mankind. Silviculture and forest management and tree breeding are disciplines that have emerged during the past two centuries, which is clearly emphasizing the unchanged importance of wood in our everyday lives. Moreover, still being a major source of energy, chemicals, and pulp, wood may appear in solid and particulate form, finding wide use for in- and outdoor applications, such as in construction, furniture, flooring, or in decorative elements. All these applications require multiscale top-down processing, utilizing stationary band saws, handheld circular saws, or high-speed vibrational sanding and wood polishing machines. These technologies have one thing in common: they all produce particulate matter. While the latter can be relatively coarse, as in the case of circular saws, huge volumes of wood dust are released from a wide range of smaller woodworking machines [1,2]. This is particularly critical for commercial indoor wood processing facilities, but also undesired in the do-it-yourself sector for two main reasons. First and most feared is that wood dust (like all organic dust) bears the risk of serious thermal events (deflagrations, explosions) occurring when a potential ignition source is present [3,4,5]. This may already happen at relatively low dust concentrations (beyond 20 g m^−3^ in air; ref. [6]), and with particles smaller than 0.5 mm [7,8,9,10]. Even if the initial event typically happens in a small compartment such as in a filter unit, or in single rooms, it is reported that sudden propagation of the reaction to neighbouring rooms or buildings may take place if the latter are connected, such as through improperly secured suction pipes. Another reason fortifying current regulatory efforts to reduce outdoor and indoor particulate pollutions is the increasing awareness of their high potential for short- and long-term health implications [1,11,12], including cardiovascular and respiratory diseases, reduced lung growth, or lung cancer [13,14], entailing an additional annual mortality rate of about 432,000 people alone in Europe (2015; [15]).

Despite considerable improvements regarding dust pollution in greater wood processing facilities accomplished by installations of costly but efficient suction and separation units (cyclones combined with filter cartridges), long-term dust exposure is still seen as a serious problem [16,17]. This is even more the case at small and medium-sized enterprises, or when it comes to the use of mobile services, i.e., smaller and hand-held processing devices [16,17]. Beyond the risk of thermal events and health issues, uncontrolled deposition of wood dust can considerably aggravate subsequent processing, as in the case of painting or varnishing. Therefore, any means capable of tackling dust formation already in statu nascendi would be highly desired.

The volumetric size of a dust cloud depends on several factors [18]. Most evidently, it is affected by the speed and type of processing tool, the size of the abrasive or cutting elements, and the feed rate and the material processed [19]. Typically, the size of a dust cloud increases inversely proportionally to the particle size, but is proportional to the dust discharge velocity [20]. Less evident but of serious impact for dust cloud formation is mechanochemical and triboelectric charging of the particles formed. This is most critical for high-speed wood processing. It has recently been shown that processing of wood and wood-based materials by circular sawing can lead to both positive particle charging, as with solid beech and spruce wood, and negative particle charging, as shown with MDF and particleboards [21]. Since the charge is locally trapped on the (large) surface of the non-conducting wood dust particles, strong repulsive forces can occur resulting in strong expansion of respective clouds and low sedimentation due to the absence of aggregation. Both effects are particularly pronounced for small, spherical, and porous particles of low density.

The theoretical base for dust charging and the occurrence of different polarity as observed for wood and wood-based materials are complex due to several interfering phenomena. According to the hot-spot theory [22,23] and the magma-plasma model [24,25], high-impact collision of the cutting elements can momentarily (<10^−7^…10^−3^ s) release very high energies at the contact points. The locally limited high energy density entails a flashing triboplasma [26], in which temperatures of up to 10,000 K, emission of excited particles (ions, radicals, low-molecular uncharged compounds), and short-term reactions may occur. Even though it has been argued that the aforementioned theories and models are not applicable for the mechano-chemistry of soft matter, the proposed pseudo-fluid model [27] and quantum-chemical calculations are also still explanatory approaches for effects hitherto not understood to a full extent [28]. Nevertheless, quantum-chemical calculations provide solid evidence that shear forces can narrow the HOMO-LUMO (which stands for highest occupied molecular orbital and lowest unoccupied molecular orbital, respectively) gap of molecules and band gap of materials, which results in destabilization of bonds up to their cleavage if the energy is high enough [29]. 

In the process of circular sawing, shear forces are supposed to play a considerable role in particle charging next to the primary cutting forces, creating new surfaces by shear tensile and compression failure of wood [4,21]. Shear forces also occur during so-called secondary particle fragmentation, which is the repeated impact of already detached particles on the cutting edge [2]. According to the pseudo-fluid model, heat transfer from the saw blade to the dust particles generated during secondary fragmentation is a factor to be considered as well as a reason for mechanochemical reactions to occur, entailing triboelectric charging to wood dust. 

The polarity generated at local surface spots of wood by processing tools, like a circular saw, cannot be described alone by triboelectric effects [28,30,31]. Triboelectric effects mainly depend on the difference in electron work function of the materials that have been brought into intimate contact for a short time. Polarity caused by triboelectric or mechano-chemical phenomena rather depends on many interfering factors. This includes the chemical nature of the friction partners, their electron affinity and mesomeric stabilization capabilities, moisture content, conductivity and extent of charge shielding, electrode potential of the metallic processing tool, and collision intensity and area as detailed above. 

Due to the poor electrical conductivity of wood, locally created surface charges can persist for longer periods of time, which finds proof in daily carpenters’ practice, where dust is found everywhere adhering on ceilings or vertical surfaces, particularly on non-conducting, oppositely charged materials. 

In the aforementioned research [21], it has been shown that circular sawing can generate both positively and negatively charged particles, depending on the material processed. These results confirm that the chemical composition of the friction partners play an important role regarding polarity and extent of charging as it is known from triboelectric series of materials abundant in literature [32].

In an own patented device (European patent EP3592465A1), it is anticipated that by volumetric splitting of a wood dust cloud and intentionally reversing the polarity of half of the dust volume, an agglomeration of oppositely charged particles takes place. This will translate into accelerated particle sedimentation, which greatly helps to reduce exposure of humans to dust and extends filter lifetime, in addition to other benefits. Reversing polarity can be accomplished by forcing wood particles into multiple intimate contact with a friction partner of higher tendency to accept or donate electrons. 

Based on the above work, here, we investigate surface coating of circular saw blades with different materials in an attempt to reduce overall particle charging by mechanochemical and triboelectric effects. This should be achieved by jointly improving sawing blade conductivity (stainless steel has poor electro-conductivity) and counteracting triboelectric charging by shear forces during secondary particle fragmentation. Aiming to relate cutting energy input to overall particle charging, cumulated machine power consumption was compared with the cumulated electric field strength that created the sawing dust.

## 2. Materials and Methods

### 2.1. Sample Preparation and Machine Setup

All experiments were conducted using beech wood (*Fagus sylvatica* L.) planks that had uniform dimensions (45 × 120 × 800 mm), dry bulk density of 691 ± 17 kg m^−^^3^, with largely uniformly orientated annual rings (ca. 45°, viewed from the face side). The planks were equilibrated at 20 °C and 65% relative humidity to give an equilibrium moisture content of 11%. Sawing of the groove was accomplished along the grain in an up-milling process using a Format4 Profil 45/07 milling machine (Felder Group, Hall in Tirol, Austria), equipped with grounded circular sawing blades of different counts of blade teeth (Albin Kraus GmbH, Tulln, Austria).

All kerfs were cut at an automatic feed rate of $v_{f}$ = 6 m min^−1^, to a final length of 800 mm, which gave a uniform processing time of eight seconds per plank. The rotation speed (n) of the saw was adapted to the count of blade teeth number (Z) according to Equation (1) to ensure uniform feed per tooth ($f_{z}$ = 0.063 mm) and largely similar particle geometry for kerfs of comparable depth (10 mm, 20 mm). To this end, the following cutting velocities were used: 45 m s^−1^ (24 teeth; equal to 4000 rpm) and 18 m s^−1^ (60 teeth, equal to 1600 rpm).

(1) $$f_{z}=\frac{v_{f}}{n\times Z}\;\;\left(\mathrm{mm}\right)$$

**Figure 1:**
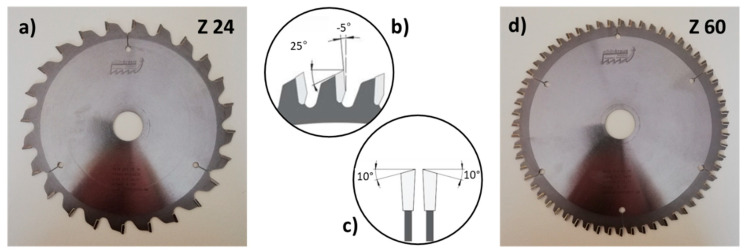
Pictures and sketches showing non-coated 24 teeth (Z24, (**a**)) and 60 teeth (Z60, (**d**)) saw blades used in this study, including geometry and arrangement of their teeth; alternating cutting edge angle was 10° (**c**); and teeth had a negative rake angle of −5°, and a back clearance angle of 25° (**b**).

The used sawing blade bodies (Figure 1) were two millimeters thick, had a diameter of 216 mm, and were equipped with alternating teeth (alternating cutting edge angle of 10°) as depicted in Figure 1c. Tooth geometry was equal for all sawing blades tested, and independent from the count of teeth. All teeth had a negative rake angle of −5°, and a back clearance angle of 25° (Figure 1b), and 2.8 mm broad cutting edges that finally resulted in somewhat broader, i.e., three millimeters wide, sawing kerfs due to blade vibrations during cutting. 

**Figure 2:**
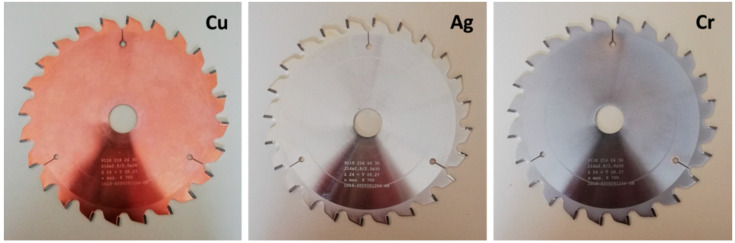
Circular sawing blades with 24 teeth and a base body made from industrial tool steel. Except for a reference blade, the base bodies were electroplated with either cupper (Cu), silver (Ag), or chromium (Cr).

Coating of the saw blade bodies with copper (Cu), silver (Ag), and chromium (Cr) was accomplished by electroplating (Vittka GmbH, Vienna, Austria), (Figure 2). An uncoated machine steel sawing blade served as reference (w/o coating, “woc”). 

Mechanochemical and triboelectric charging is primarily a surface phenomenon due to the poor electro-conductivity of wood. Therefore, surface-to-volume ratio of particles generated would be an important parameter to monitor. However, owing to the immense difficulties related to dust surface analysis, the electric field strength of comparable volumes of wood dust produced upon cutting of the grooves at uniform feed per tooth ($f_{z}$ = 0.063 mm) and cutting time (8 s), respectively, were measured instead. Since the cutting kerf had a depth of either 10 mm or 20 mm and uniform width of 3 mm, the released volumes of wood dust used as reference for electric field strength and power analysis were 24 and 48 cm^3^, respectively. 

**Figure 3:**
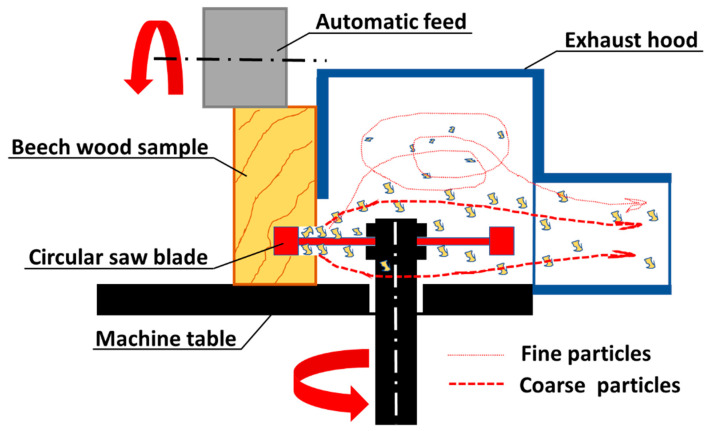
Milling machine with circular saw blade for the wood dust generation.

**Figure 4:**
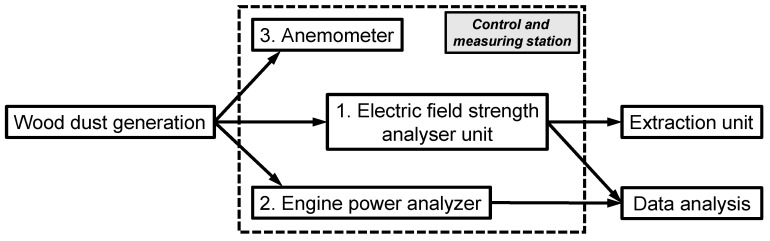
Experimental control and measuring setup.

A grounded APU 300 air extraction unit (AL-KO THERM GmbH, Jettingen-Scheppach, Germany) was connected to the circular saw via an exhaust hood (Figure 3) for quantitative collection of the dust produced and feeding the field mill for electric field strength measurements (Figure 3 and Figure 4). A constant airflow velocity of 25 m s^−1^ was maintained, this was continuously monitored by means of a HVAC 2 anemometer (PCE Instruments, Meschede, Germany). Figure 4 shows a schematic of the experimental control and measuring setup comprising anemometer, electric field mill (EFM), and saw power consumption recorder. Wood dust for particle size analysis was collected separately using a mobile R200TE air extraction unit consisting of cyclone and downstream dual chip cartridge filter (6.5 m^2^ filter area, Holzprofi Pichlmann GmbH, Roitham, Austria).

### 2.2. Power Analysis in the Cutting Process

Electrical power consumption *P* of the circular saw was determined for each of the produced grooves in time intervals of two seconds using a PA 8000 three-phase power data logger (PCE Instruments, Meschede, Germany). Based on the electric current that is measured for each of the three phases using a set of current sense jaws, the PA 8000 instrument calculates the electrical power automatically by multiplying the recorded currents *I*_1_ to *I*_3_ with the respective voltages *U*_1_ to *U*_3_ measured simultaneously between each phase and the neutral wire (Equation (2)).

(2) $$P_{t}={\left(U_{1}\cdot I_{1}\right)}_{t}+{\left(U_{2}\cdot I_{2}\right)}_{t}+{\left(U_{3}\cdot I_{3}\right)}_{t}\;\;\;\;\;\left(t=1\ldots 13\right)$$

The respective machine power consumption values expressed in watt-second (W s, seven measurement values per cut) were subsequently used to calculate the average total energy *E_cut_* (J) required for completing sawing of one groove, which was finally normalized to one cubic centimeter of wood released (J cm^−3^).

### 2.3. Field Strength Detection and Accumulated Triboelectric Particle Charge

The electric field strength, i.e., the force field created by the entire collective of charged particles relative to ground was measured semi-continuously by using a field strength analyzer unit (Figure 4). This unit consists of a cylindrical measuring module (inner diameter 150 mm, length 200 mm) that was inserted at a distance of 300 mm from the sawing blade into an 800 mm long horizontal section of the exhaust pipeline. The measuring module is equipped with a MK11 gauge head connected to an EFM-115 electrical field mill (resolution 5 V m^−1^; Kleinwächter GmbH, Hausen, Germany), both mounted onto the exterior of the insulated measuring module. The latter was used to measure the net electric field strength of the saw dust in the exhaust stream in one-second intervals. Before each cut the module was grounded, “taring” the field strength analyzer.

The electric field measured inside the insulated cylindrical measuring module is caused by charged wood particles traveling through the exhaust line. While it is assumed that most of the particle charging occurs during the cutting process, minor contributions by triboelectric charging upon collision of dust particles with the pipe surface before entering the measuring module cannot be fully excluded. Since cutting with the differently surface-coated saw blades was conducted in the same manner, i.e., uniform feed per tooth and cutting time, the count of particle collisions can be assumed to be similar. This is at least the case for kerfs of the same depth. However, it is worth noting that the two different kerf depths investigated require separate consideration. Since particle size increases with cutting depth even at constant feed per tooth [33], this suggests that less charging should occur for the deeper cuts. Since tribomechanical and triboelectrical charging is mainly a surface phenomenon due to the poor electrical conductivity of wood, maintaining a constant surface to volume ratio was envisaged. It was realized by adjusting the cutting parameters in such a manner that feed per tooth and therefore particle size was theoretically independent from the number of teeth.

### 2.4. Particle Size Proportions

Wood dust produced at constant feed rate of 12 m min^−1^ and a cutting depth of 20 mm was collected using the above-described mobile air extraction unit. The material collected from the upstream cyclone was split into four portions (40 g each) that were independently subjected to particle size analysis using a two-stage AS 300 Control cascade impactor (Retsch GmbH, Haan, Germany). The latter was operated at 80% amplitude and a time period of 30 min which allowed the material to separate into the following fractions: x > 250 μm, 250 ≥ x ≥ 100 μm, x < 100 μm. Since only a negligible amount of wood dust was found in the downstream cartridge filter, comparable measurements using the cascade impactor were not feasible.

The results of each stage of the cascade impactor are presented as the mean value of weight percentage (*n* = 4). Since the cutting parameters are different for dust analysis and the investigations related to the impact of saw blade coating on mechanochemical/triboelectric charging, electric field formation, and energy consumption, particle size analysis has orienting character only.

### 2.5. Experimental Design and Statistics

The three experimental variables of this study comprise (i) the type of saw blade base body plating (untreated reference without coating “woc”, copper, chromium, and silver), (ii) the count of saw teeth per circular saw blade (24, 60), and (iii) the depth of the targeted saw groove (10, 20 mm). Single-step parameter variation was accomplished with eight repetitions, which concomitantly represents the number of repetitions of the field strength measurements. Aiming to keep differences with regard to wood particle size and geometry as low as possible for the settings tested, saw blade rotation speed was normalized for the count of saw teeth to ensure uniform feed per tooth. Thus, rotation speeds of 4000 rpm (24 teeth blades) and 1600 rpm (60 teeth) were chosen. Electric field strength (kV m^−1^) measured in intervals of 1 s was related to the released volume of saw dust (expressed in kV m^−4^), which allowed a comparison of the net electric charges created by the different test settings. Average power consumption values were multiplied with the time required to cut one groove (8 s), yielding the net energy consumed (kWh). Conversion of this value into the SI unit Joule (1 kWh = 3.6 × 10^6^ J) and relating it to the dust volume produced per cut (J cm^−3^) represented the theoretical upper limit of energy transferred to the dust particles. This is of course a theoretical number only since multiple energy losses occur during cutting, such as in the form of thermal, vibrational, and excitation energy. However, by connecting electric power consumption normalized for a defined volume of dust produced with the extent of triboelectric dust charging as measured by an electric field mill, the influence of sawing parameters on dust cloud characteristics could be elucidated.

Statistical analysis was carried out using SPSS 24 (IBM, USA). Descriptive statistics were presented in form of boxplots to facilitate visual perception of the effects of the different parameters tested. Unpaired *t*-tests were employed for the particle size data. Analyses of variance (ANOVA) were conducted to compare the influence of the different variables on both electric field strength and energy consumption. Homogeneity of variance was assessed with the Levene’s test. Variance homogeneity for internal groups was assessed using the Bonferroni post hoc test.

## 3. Results and Discussion

### 3.1. Particle Size Proportion

**Figure 5:**
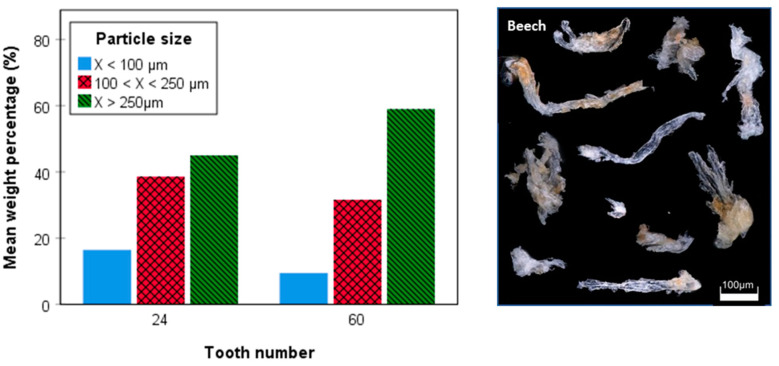
Particle size proportion of tooth number 24 and 60 for beech wood showing a significant difference (*t*-test, *p* < 0.05, *n* = 4; (**left**)); microscopic picture of the produced dust particles (**right**).

Particle size analysis of saw dust produced at a constant feed rate of 12 m min^−1^ revealed that at otherwise comparable conditions (feed per tooth, cutting depth, etc.), the 24 teeth blade yielded significantly higher (sig. *p* < 0.05) weight fractions of particles < 100 as well as 100–250 µm, compared with the 60 teeth blade (Figure 5). This difference was even more pronounced for the particle fraction < 100 µm, where twice the amount of dust was produced with the blade having fewer teeth (Figure 5). Being in good agreement with a recent study [2], these findings suggest that secondary particle fragmentation in the narrow gaps between saw blade and the solid wood is here more pronounced, affording finer particles as originally produced during primary cutting. This is likely caused by the higher rotational speed of the saw blade 24 teeth (8000 rpm vs. 3200 rpm for the blade 60 teeth), which was chosen to ensure uniform feed per tooth for cutting of the grooves. This is supported by findings demonstrating that sawdust of equal particle size is obtained when beech wood planks are cut in longitudinal direction at uniform rotation speed (800 rpm) using blades of 24 and 60 teeth, respectively [21].

According to the wisdom words “the dose makes the poison” by the medieval doctor, alchemist, and natural philosopher Theophrastus Bombast von Hohenheim (Paracelsus, 1493–1541), quantitative considerations are important to assess the health risk of wood dust released during wood processing. At a slow manual feed rate of about 1 m min^−1^ as in the work of Myna et al. [21], cutting of a 120 mm long groove (depth 20 mm, 7 s) into beech wood planks releases a wood volume of approximately 7.2 cm^3^ in the form of a voluminous dust cloud with particles precipitating within different periods of time. In the presented work, the released amount of wood dust volume was 4.5 times higher (33 g), due to the larger cutting length (800 mm) and time (8 s). Projected on an hourly output and assuming 30 min of continuous sawing per hour, approximately 7440 g or 10,800 cm^3^ of wood dust would be produced. Even if it can be taken for granted that most particles would be captured by respective exhaust units, the remaining fraction would spread across the surrounding air, as is frequently the case in carpentry shops, or with mobile parquet sanding machines. For healthcare reasons, this non-captured dust fraction needs to be set in context of the permitted emission limits and workplace concentrations as detailed in the Austrian Limit Value Ordinance [34]. According to these regulations, the wood dust concentration must not exceed a concentration of 2 mg m^−3^, which is just a tiny fraction of the hourly dust production of the above assumed scenario. It is even more critical than it might appear at first glance, since particles smaller than about 50 µm are invisible for most people and sediment very slowly.

**Figure 6:**
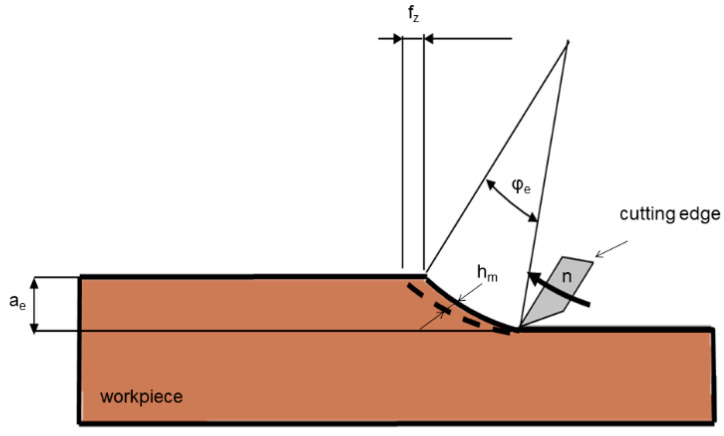
Schematic presentation of the parameters required for the calculation of chip thickness *h_m_* (Equations (3) and (4)) and chip arc length s_b_ (Equation (5)).

According to theory, a homogeneous material with high toughness, such as polycarbonate, should have a theoretical mean particle (chip) thickness of $h_{m}$ = 20 µm, and a length of $s_{b}$ = 67 mm, independent of the number of teeth Z (Equations (3)–(5)). However, as evident from data shown in Figure 5, much smaller particles barely exceeding a length of 0.4 mm were obtained, which clearly confirms that the Equations (3)–(5) in Figure 6 cannot be applied to inhomogeneous and fibrous materials like wood, since fiber orientation and secondary particle fragmentation has a strong additional upgrinding effect. Furthermore, the data show that despite adaption of the saw rotational speed aiming to ensure equal feed per tooth ($f_{z}$, Equation (1)), distinct differences with regard to particle size distribution were obtained for the blades of different toothing. In Equations (3)–(5), further details are *a_e_* being the cutting depth, *d* the tool diameter, φ the pressure angle, *v_f_* the feed rate, and *r* the tool radius.

(3) $$h_{m}=\;\frac{f_{z}*a_{e}*360}{d*\pi *\varphi }$$

(4) $$h_{m}=\;\frac{\frac{v_{f}*1000}{n*Z}*a_{e}*360}{d*\pi *\cos^{-1}\frac{r-a_{e}}{r}}$$

(5) $$s_{b}=d*\pi \;\frac{\varphi \;}{360}$$

### 3.2. Power Analysis during Sawing

According to the first law of thermodynamics, the total energy of an isolated system comprising the two kinds of energy transfer, i.e., heat and thermodynamic work, is constant. However, all wood processing technologies represent open systems from the thermodynamic point of view, characterized by losses of electric energy through conducted mechanical work, heat, or even (ultra)sound. It is assumed that a significant fraction of energy consumed during cutting is responsible for the generation of charged surfaces by mechanochemical and triboelectric phenomena. The extent of charge generation is presumably much higher for the particulate material expelled from the progressing groove than for the processed solid wood. This is caused by several factors including secondary particle fragmentation and the unparalleled larger surface of wood dust compared to the groove surface and less pronounced charge neutralization by the rotating sawing blade.

Aiming to reduce charging of wood dust, surface coating of the circular saw blades by plating with metals (Ag, Cu, Cr) of different electro-conductivity and different position in the triboelectric series of materials was conducted. It was argued that this facile measure would improve sawing blade conductivity to neutralize potential differences (stainless steel has poor electro-conductivity) and counteract triboelectric charging, e.g., due to secondary particle fragmentation.

**Figure 7:**
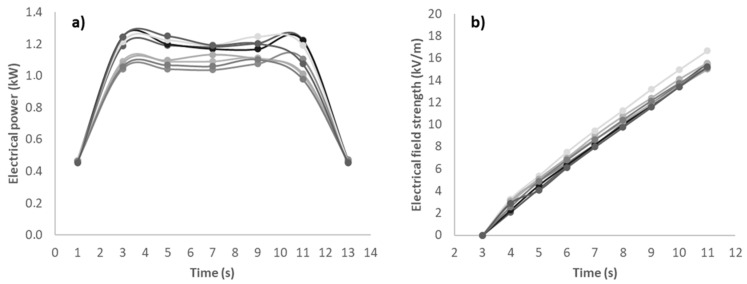
Machine power consumption (**a**) and development of cumulated electrical field strength ((**b**), plateau phase only) during cutting of an 800 mm long and 10 mm deep grooves into 8 beech wood panels using a w/o coated 60 teeth circular sawing blade (1600 rpm, *f_z_* = 0.063 mm), *n* = 8.

Aiming to evaluate the effect of the different coatings on energy losses by friction, the net energy *E_cut_* (J) consumed for cutting of 800 mm long grooves into beech planks was compared for two cutting depths (10, 20 mm) and two sawing blades (24, 60 teeth) at the above detailed conditions. For this purpose, machine power consumption was discontinuously measured in idle state (*t* = 0) and every two seconds during cutting until the saw blade entirely left the plank (Figure 7). Further electrical power and electrical field strength data for different coatings, groove depths, and frequency of rotations (rpm) are shown in Figures S1–S4. 

In idle state, power consumption of the circular saw was virtually unaffected by both plating and count of cutting edges. The different rotational speed adjusted to ensure equal feed per tooth just gave rise to a marginally higher starting energy for the Z 24 blade (5700 J) compared to its Z 60 counterpart (4650 J). Electric power consumption *P* (kW) rose strongly with the onset of the cutting process to reach a largely constant plateau value after two seconds. This plateau was then largely maintained until the load start to decline towards the end of cutting, which was after some eight seconds.

It is well-known that the power consumption *P* is a function of cutting depth, rotational speed, type of plating, and the extent of sample inhomogeneities. The latter comprise variations in arrangement of annual rings, fiber orientation, or presence of knots, all potentially giving rise to local inhomogeneities in composition, density, and hardness. Aiming to offset fluctuations in *P* by the above impact factors, eight independent power consumption curves were recorded (i.e., grooves were cut) per variant. The average total energy *E_cut_* consumed per cutting of one groove was then calculated for each of the tested variants by adding up the average *P* values of every two consecutive measurements, multiplying them with time elapsed until the next data were recorded (2 s) and converting *E_cut_* into the SI unit Joule (1 kWs = 1 × 10^3^ J; Equation (6)).

(6) $$E_{cut}=\;\frac{P_{1}+P_{2}}{2}\cdot ∆t+\frac{P_{2}+P_{3}}{2}\cdot ∆t+\frac{P_{3}+P_{4}}{2}\cdot ∆t+\frac{P_{4}+P_{5}}{2}\cdot ∆t+\frac{P_{5}+P_{6}}{2}\cdot ∆t+\frac{P_{6}+P_{7}}{2}\cdot ∆t$$

Normalization of *E_cut_* for one cubic centimeter of wood released during cutting (*E*_V_ in J cm^−3^) of the grooves was accomplished in an attempt to express the maximum energy transferred to dust and responsible for electrostatic charging, which is most likely responsible for the repulsive forces between the particles and consequently for long-term persistence of dust clouds. It could of course be a theoretical number only, since multiple energy losses occur during cutting, as described earlier.

**Figure 8:**
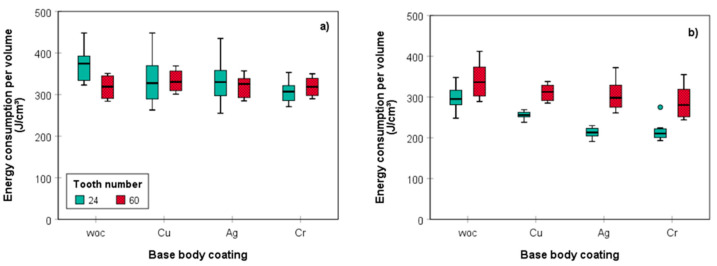
Energy consumption in joule per cubic centimeter of produced dust for (**a**) T10 and (**b**) T20, *n* = 8.

The data compiled in Figure 8 confirm that both the different revolutions per minute, chosen to maintain uniform feed per tooth, and the different cutting depths give rise to different *E*_V_ values. While cutting depth had a marginal impact on *E*_V_ for the Z 60 blade, obviously due to the slower rotation speed (1600 rpm), the differences were more pronounced for its Z 24 counterpart (sig. *p* < 0.05). Here, the value of *E*_V_ for cutting of the 20 mm deep groove with the uncoated blade was almost 25% lower (298 J cm^−3^) compared to the kerf of 10 mm depth (372 J cm^−3^). This observation is in accordance with the literature and has been traced back to the lower effective cutting force [33]. This is due to both the extent of particle splitting and size of frictional area between sawing blade, wood, and released particles, which increases with cutting depth. As a result, particle size increases with cutting depth along with the stiffness of individual particles. Abrasion can be neglected in these considerations, since only new sawing blades were used which were replaced after cutting of 12.8 m (16 planks).

The stronger variation in *E*_v_ observed for the Z24 saw blade is assumed to be a result of the lower cutting force. It is known from earlier studies that cutting force depends on various factors, such as wood density [35] and moisture content [36]. As these parameters were largely held constant in this study, cutting speed requires a closer consideration. According to Gottlöber [33], the cutting force minimum is reached at a cutting speed of 40 m s^−1^, which is thought to be related with pre-splitting. Below and above this critical cutting speed, forces are increasing. If the cutting speed is higher than the speed of the pre-splitting, it can no longer be used, which means that more energy has to be used for disintegration of wood. Additionally, more energy is used for particle acceleration at higher speeds. This is in good agreement with the observed results revealing that using the Z24 blade at 45 m s^−1^ cutting speed, less energy was required compared to the Z60 blade operated at 18 m s^−1^. This is particularly evident for cutting of the 20 mm deep grooves where the difference could be seen for all uncoated and plated saw blades, while the differences were rather marginal for shallow kerfs.

The high sensitivity of the variant featuring the least cutting force (20 mm deep groove, blade 24 teeth) towards parameter variation allows for tracing back the impact of the different saw blade coatings with metals of different position in the triboelectric series of materials. The data in Figure 8 (Z24, T20) confirm that all plating variants and increasing in the order copper < silver ≈ chromium are capable of reducing *E*_v_, i.e., the energy required to convert 1 cm^3^ of solid wood into dust. This is a particularly interesting observation as it provides evidence of a further reduced cutting force, probably fostered by reduced friction. At this point, it is difficult to provide a satisfying explanation for this surprising finding; however, enhanced heat conductivity and electric conductivity of the different metallic coatings interfering with triboelectrical phenomena during secondary particle fragmentation are some of the likely reasons.

### 3.3. Triboelectric Charging of Dust Particles from Solid Beech Wood

Wood dust particles can be largely heterogeneous in terms of size, shape, aspect ratio, surface area, porosity, and surface roughness, depending on the processing technique employed (see also Figure 5 right). This renders continuous measurements of surface charges generated by triboelectric and tribomechanic processes—specifically in status nascendi—highly challenging, if not impossible. It is aggravated by the fact that charge density on the surface of wood particles can be quite heterogeneous due to local differences in impact energy and the poor electro-conductivity of dry wood. Therefore, the net electric field strength $\vec{E}$, i.e., the force field created by the entire collective of charged particles relative to ground, was semi-continuously measured (one second intervals, kV m^−1^) in the exhaust line, cumulated, and related to the volume of wood released in the form of dust during cutting of the 10 mm (24 cm^3^) and 20 mm (48 cm^3^) deep grooves (${\vec{E}}_{t,V}\;$ in kV m^−4^).

**Figure 9:**
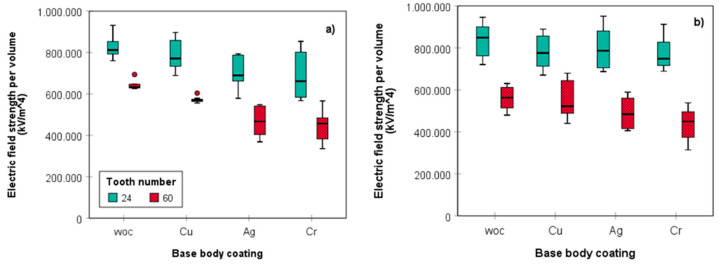
Electric field strength of wood dust produced by circular sawing for (**a**) T10 and (**b**) T20; woc = uncoated, *n* = 8.

The data in Figure 9 clearly show that plating of the saw bodies with the different metals reduced the extent of triboelectric charging. This is particularly pronounced for cutting with the blade 60 teeth, independent of the cutting depth. While plating with copper entailed here a reduction of cumulated field strength of about 10.8–13.8%, these values dropped significantly (sig. *p* < 0.05) for silver (27.2–27.8%), but most prominently for chromium coated blades (30.9–32.0%), with the somewhat higher values always obtained for the deeper kerfs (Table S2, for energy consumption also Table S1). Independent of both depths of the grooves and number of teeth (rotations per minute), the same trend was observed for all variants, i.e., cumulated field strength decreased in the order woc > Cu > Ag > Cr. This confirms the initial hypothesis that by intimate contact of wood dust with friction partners of different positions in the triboelectric series of materials, charging can be controlled to a certain extent. The selection of the metals tested here was based on an own experimental triboelectric series of elements with copper being closest, silver intermediary, and chromium furthest away from steel, an order that is well reflected by the order of volume normalized decreasing electrical charging of dust particles. However, considering that the driving forces responsible for the phenomenon of triboelectric charging are hitherto still far from being resolved and the subject of intensified research [32,37], it is still too early to draw general conclusions as many other factors caused by the different coating materials can have an impact, too. This includes differences in surface roughness, heat release, electrical conductivity, hardness, strength, abrasion-resistance, and resistance to surface oxidation which can be quite pronounced as evident just from a comparison of two of the plated metals, i.e., copper and chromium [38].

Nevertheless, plating of the circular sawing blades with chromium turned out to be the most efficient coating as it reduced not only machine power consumption by more than 20% for 20 mm deep grooves but also significantly impeded electrical charging of wood dust particles. This effect was most pronounced for sawing of the shallow grooves (10 mm) using the Z60 sawing blade. The median values of ${\vec{E}}_{t,V}$ followed the same trend, however, the extent of charge reduction was clearly lower for this variant.

It is worth noting that independent of the coating and cutting depth, a considerable reduction of up to 40% related to cumulated electric field strength and, hence, of electrical dust surface charging can be achieved if blades with 60 teeth are used instead of those with 24 teeth. This is mainly due to the lower revolutions per minute (1600 rpm instead of 4000 rpm) required to provide the same feed per tooth (*f_z_* = 0.063 mm). This obviously reduces the extent of tribomechanical and triboelectrical charging during the cutting process and consequently, in the course of secondary particle fragmentation, the weight fraction of fine particles < 250 µm and hence the overall particle surface area. We therefore can approve the stated hypothesis saying that surface coating of circular saw blades aims at a reduction of wood particle charging through mechanochemical and triboelectric charging effects. Concomitantly, the contact time between charged particles and sawing blade is prolonged, which could entail two interfering effects, i.e., improved discharging of wood dust by grounding and partial charge reversion by friction between wood particles and the respective coating material. The obtained results also suggest that the ratio between wood–wood friction and wood–saw blade friction depends on the cutting depth. Doubling of the dust volume increases the probability of wood particles interacting with each other (secondary friction) at the expense of intimate contacts with the metal surface (primary friction).

Recent experiments using an entirely different milling setup have shown that isolation of both milling chamber and metal coated cutting device is able to reverse polarity and impart wood dust negative polarity. Investigations aiming to study the development of field strength caused by wood dust in exhaust lines when an isolated circular sawing blade is used, will be subject of future work.

## 4. Conclusions

It has been shown with the example of beech wood planks that machine power consumption normalized for one cubic centimeter of removed wood was higher for the saw blades with 60 teeth than for the blades with 24 teeth, even though the rotation speed was significantly lower (18 m s^−1^ vs. 45 m s^−1^) to ensure uniform feed per tooth. This is indicative of a lower friction resistance at high rotation speeds, which becomes even more evident when increasing cutting depth to 20 mm. Except for cutting of the 20 mm deep grooves using a chromium plated saw blade 60 teeth, virtually no link between machine power consumption and electrostatic charging of sawdust was found. However, it was demonstrated that the extent of electrostatic sawdust charging can be manipulated by the type of friction partner used to coat the sawing blade base bodies. Coating with chromium turned out to be most effective in shifting the electrostatic charge of the wood particles towards neutralization. Lowering of rotation speed using circular saw blades of higher toothing is an additional measure significantly reducing the electrostatic charging of wood dust.

## 5. Patents

The idea of utilizing opposite electrostatically charged wood particles for dust control went down in the European patent EP3592465A1. A second patent (pending) A50217/2020 (523614) is directly linked to this published work.

## Data Availability

The data presented in this study are available on request from the corresponding author.

## Supplementary Materials

The following are available online at https://www.mdpi.com/article/10.3390/ma14185123/s1. Figure S1: Machine power consumption (kW) for cutting of an 800 mm long and (a,c,e,g) 10 or (b,d,f,h) 20 mm deep groove into beech wood panels using 60 teeth circular sawing blades (1600 rpm, *f_z_* = 0.063 mm), uncoated (woc; a,b), or coated with Cu (c,d), Ag (e,f), and Cr (g,h), Figure S2: Machine power consumption (kW) for cutting of an 800 mm long and (a,c,e,g) 10 or (b,d,f,h) 20 mm deep groove into beech wood panels using 60 teeth circular sawing blades (1600 rpm, *f_z_* = 0.063 mm), uncoated (woc; a,b), or coated with Cu (c,d), Ag (e,f), and Cr (g,h), Figure S3: Electrical field strength (kV/m) for cutting of an 800 mm long and (a,c,e,g) 10 or (b,d,f,h) 20 mm deep groove into beech wood panels using 60 teeth circular blades (1600 rpm, *f_z_* = 0.063 mm), uncoated (woc; a,b), or coated with Cu (c,d), Ag (e,f), and Cr (g,h), Figure S4: Electrical field strength (kV/m) for cutting of an 800 mm long and 10 mm (a,c,e,g) or 20 mm (b,d,f,h) deep groove into beech wood panels using 24 teeth circular sawing blades (4000 rpm, *f_z_* = 0.063 mm), uncoated (woc; a,b), or coated with Cu (c,d), Ag (e,f), and Cr (g,h), Table S1: Descriptive statistics of energy consumption in joule per cubic centimeter of produced wood dust, Table S2: Descriptive statistics of energy consumption in joule per cubic centimeter of produced wood dust.
