# Supplementary material for: Can Surface Coating of Circular Saw Blades Potentially Reduce Dust Formation?
Source: Materials (Basel). 2021 Sep 7;14(18):5123. doi: 10.3390/ma14185123 (PMC8469391; doi:10.3390/ma14185123)
Supplement: Supplementary file 1 [file materials-14-05123-s001.zip › materials-1329402-supplementary.pdf]

materials

MDPI

Supplementary Information

Can Surface Coating of Circular Saw Blades Potentially Reduce Dust Formation?

Roman Myna [1,2], Raphaela Hellmayr [1], Maria Georgiades [1], Lena Maria Leiter [1], Stephan Frömel-Frybort [2], Rupert Wimmer [1,*] and Falk Liebner [3]

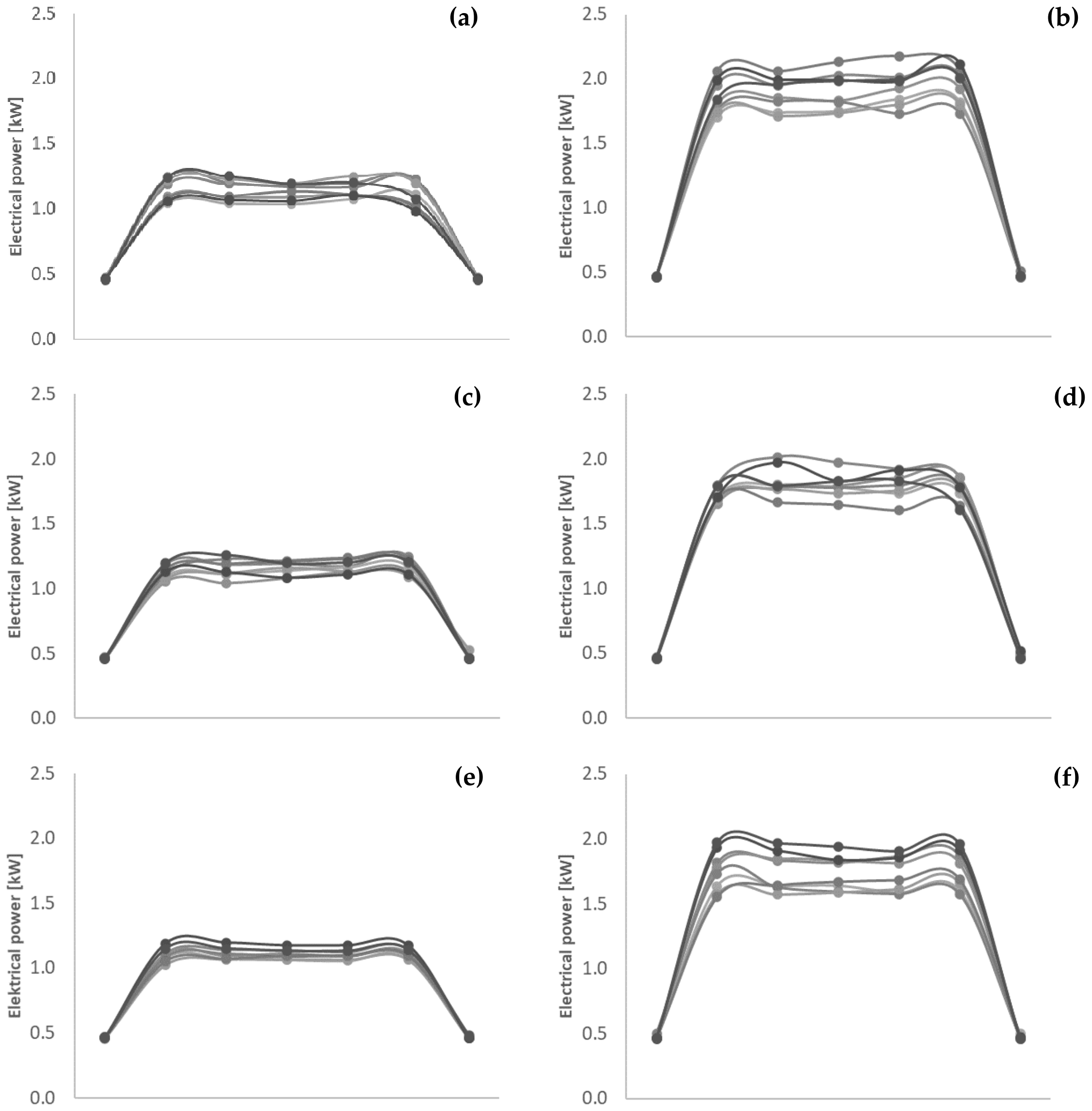

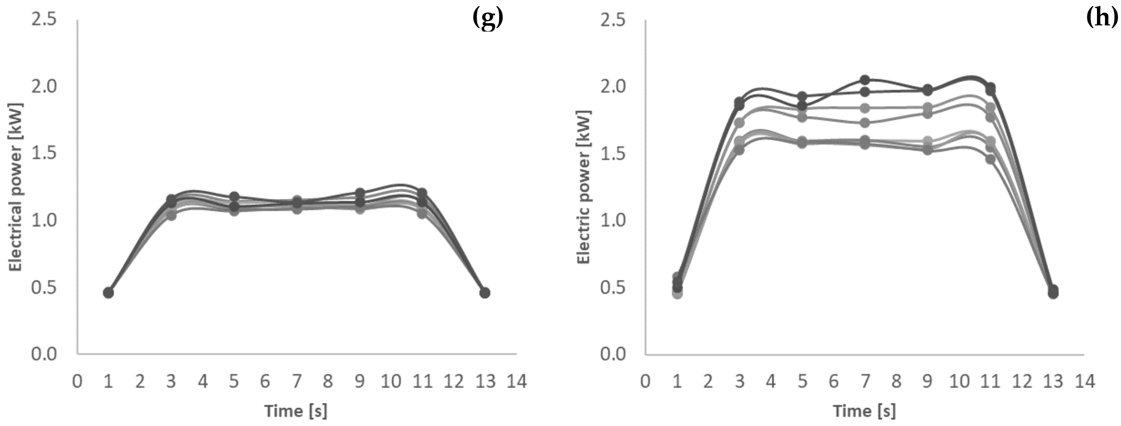

Figure S1. Machine power consumption (Kw) for cutting of an 800 mm long and (**a**, **c**, **e**, **g**) 10 or (**b**, **d, f, h**) 20 mm deep groove into beech wood panels using 60 teeth circular sawing blades (1.600 rpm, f_z = 0.063 mm), uncoated (woc; **a**,**b**) or coated with Cu (**c**,**d**), Ag (**e**,**f**) and Cr (**g**,**h**).

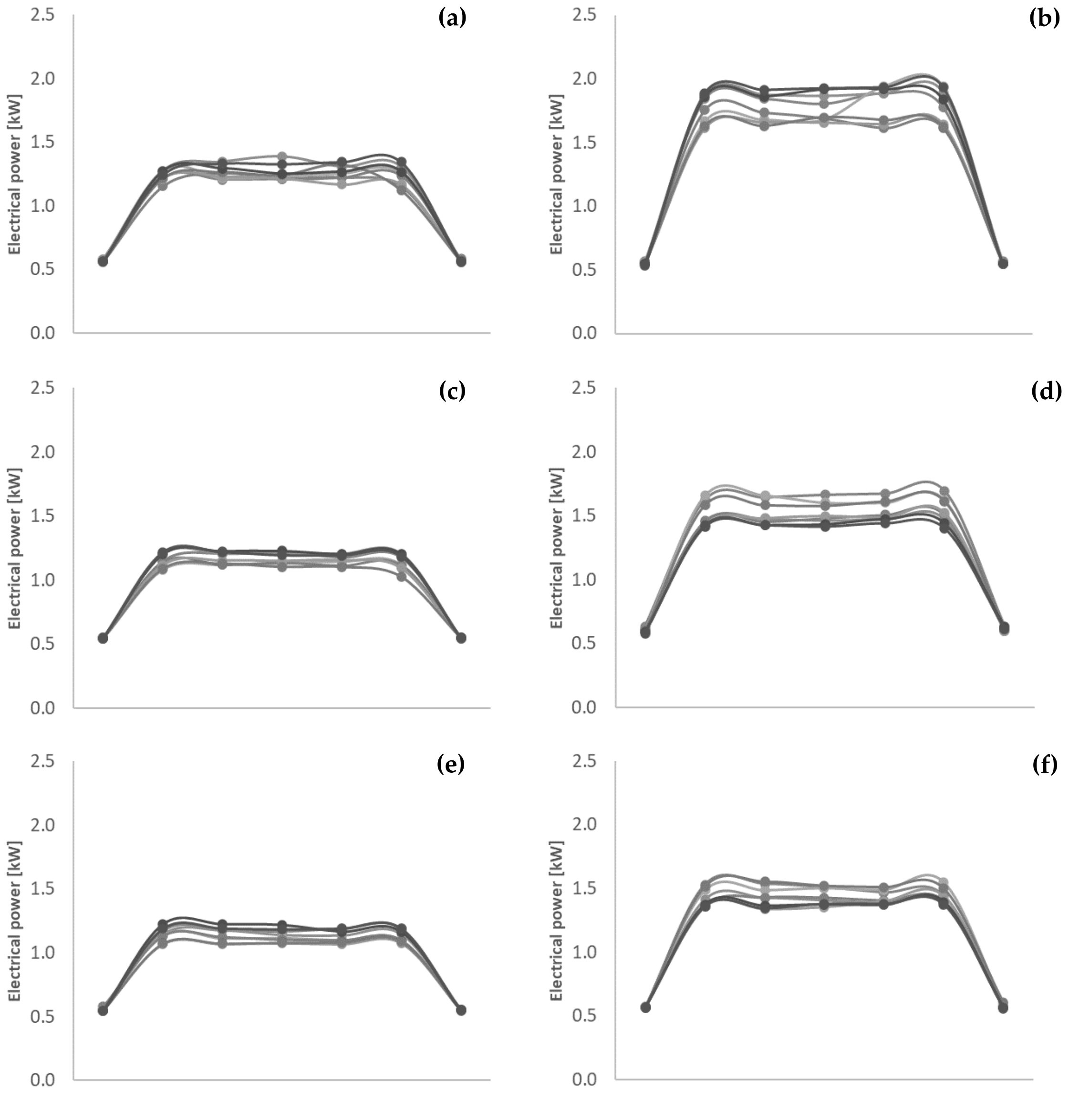

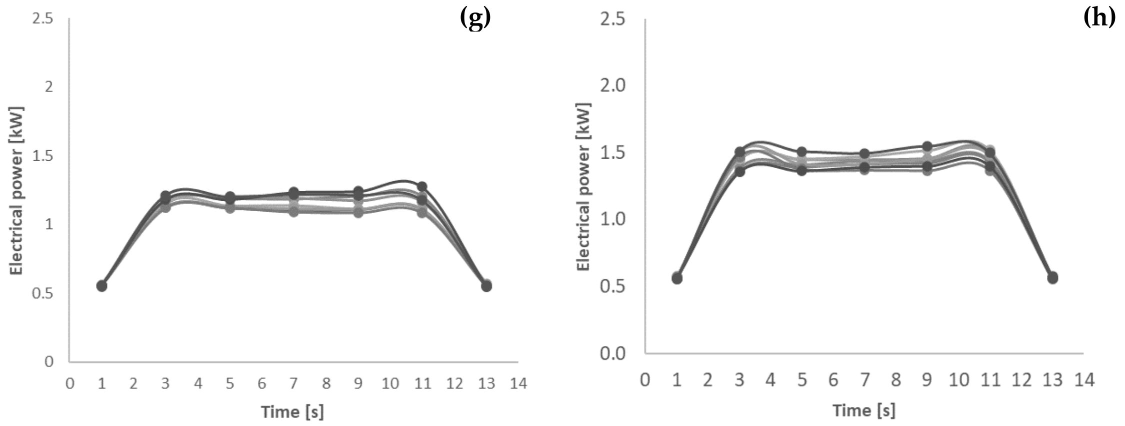

Figure S2. Machine power consumption (Kw) for cutting of an 800 mm long and (**a**, **c**, **e**, **g**) 10 or (**b**, **d, f, h**) 20 mm deep groove into beech wood panels using 24 teeth circular sawing blades (4.000 rpm, f_z = 0.063 mm), uncoated (woc; **a**,**b**) or coated with Cu (**c**,**d**), Ag (**e**,**f**) and Cr (**g**,**h**).

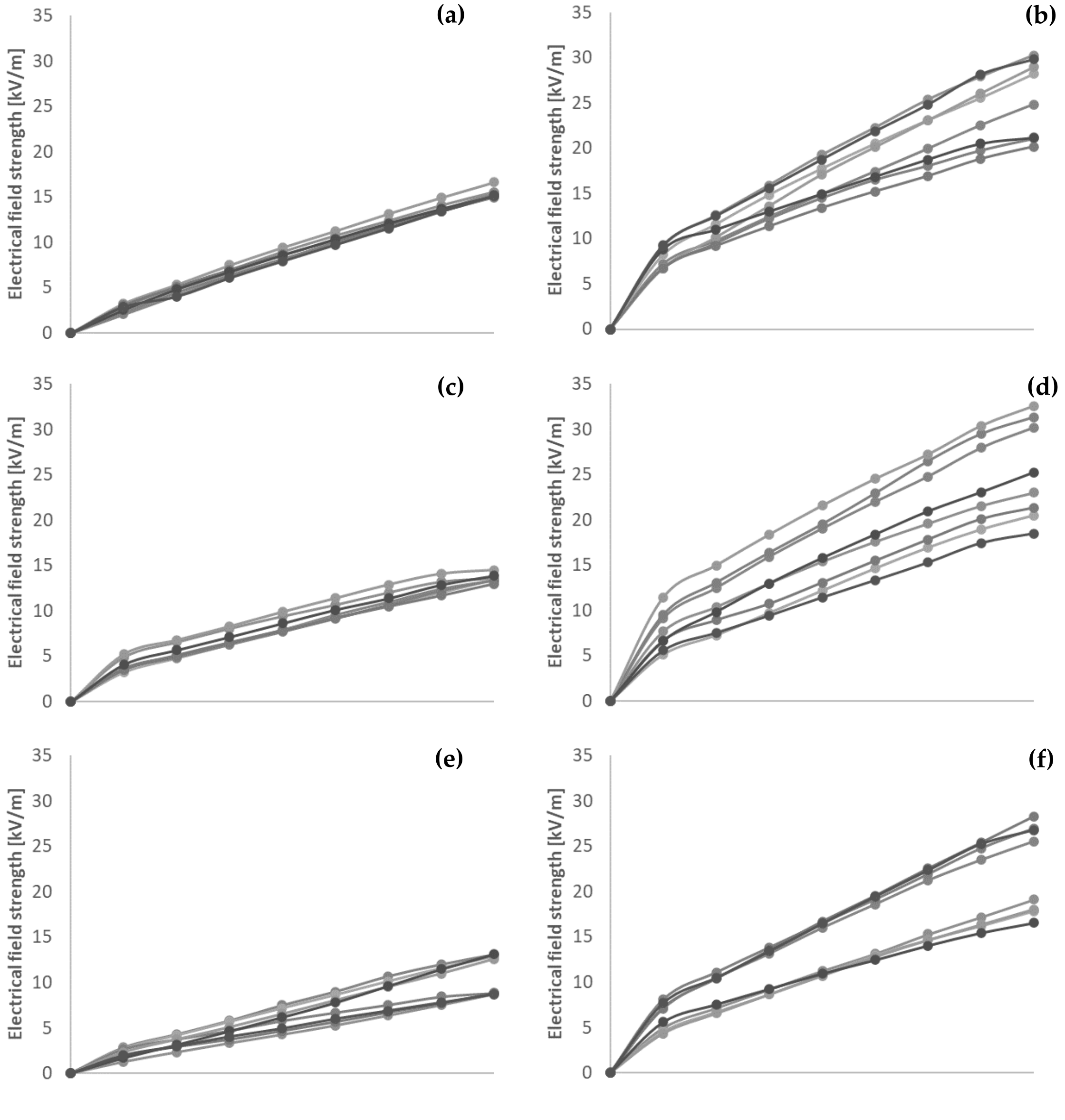

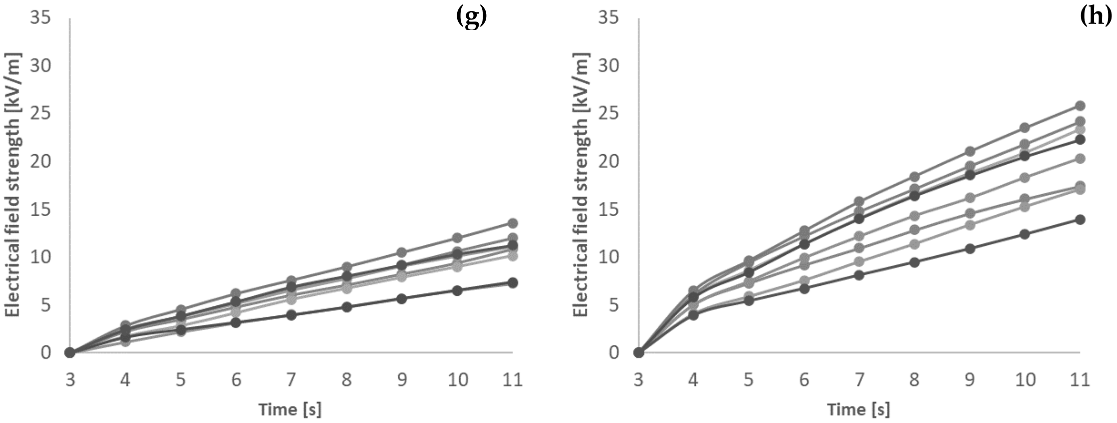

Figure S3. Electrical field strength (kV/m) for cutting of an 800 mm long and (**a, c, e, g**) 10 or (**b, d, f, h**) 20 mm deep groove into beech wood panels using 60 teeth circular blades (1.600 rpm, f_z = 0.063 mm), uncoated (woc; **a**,**b**) or coated with Cu (**c**,**d**), Ag (**e**,**f**) and Cr (**g**,**h**).

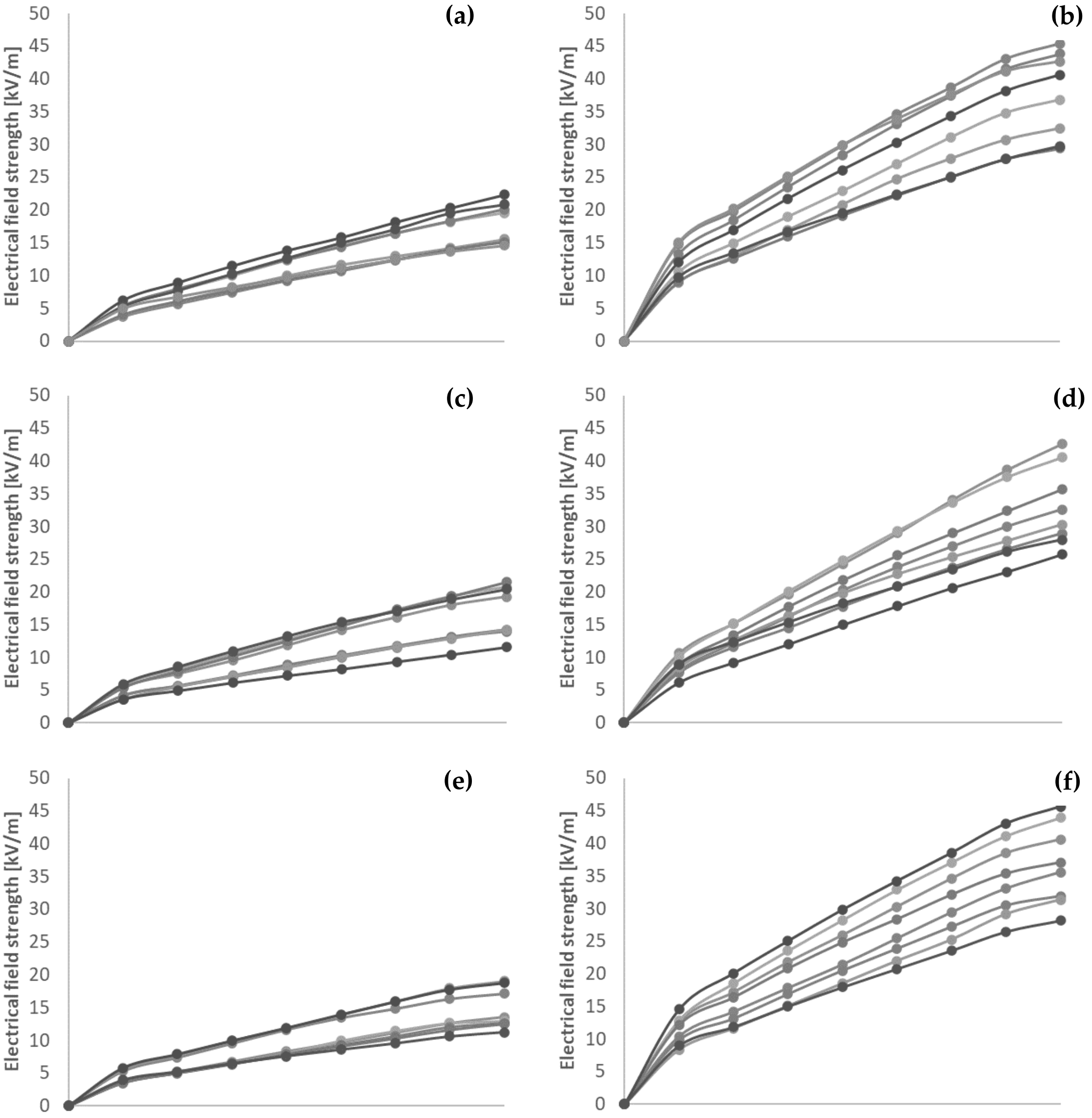

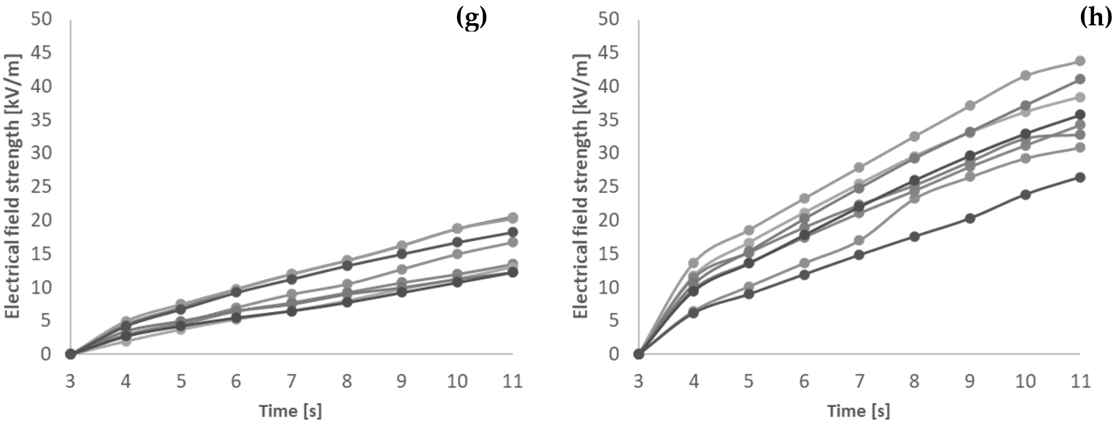

Figure S4. Electrical field strength (kV/m) for cutting of an 800 mm long and 10 mm (**a, c, e, g**) or 20 mm (**b, d, f, h**) deep groove into beech wood panels using 24 teeth circular sawing blades (4.000 rpm, f_z = 0.063 mm), uncoated (woc; **a**,**b**) or coated with Cu (**c**,**d**), Ag (**e**,**f**) and Cr (**g**,**h**).

Table S1. Descriptive Statistics of energy consumption in Joule per cubic centimetre of produced wood dust.

		**Energy consumption (J/cm³)**			
		10 mm deep groove		20 mm deep groove	
coating	Teeth number	Mean	σ	Mean	σ
woc	24	371.63	41.93	297.62	30.92
	60	318.13	29.64	340.75	45.24
Cu	24	335.50	61.38	255.88	9.67
	60	332.88	26.65	311.00	20.34
Cr	24	306.50	27.41	216.75	26.01
	60	318.88	22.78	287.63	42.14
Ag	24	332.62	55.13	212.75	13.16
	60	319.50	26.79	304.63	37.42

Table S2. Descriptive Statistics of electric field strength of wood dust produced by circular sawing.

		**Electric field strength (kV/m⁴)**			
		10 mm deep groove		20 mm deep groove	
coating	Teeth number	Mean	σ	Mean	σ
woc	24	826 042	53 446	836 207	82 297
	60	642 188	22 327	561 009	56 901
Cu	24	789 086	75 737	781 332	83 764
	60	572 581	15 767	553 796	89 827
Cr	24	689 670	118 200	773 365	77 079
	60	443 895	77 156	436 484	76 810
Ag	24	706 723	77 546	797 982	99 546
	60	467 824	77 443	489 423	78 165
